# 局部晚期中央型肺癌化疗联合免疫新辅助治疗后机器人辅助支气管袖状切除的安全性与可行性

**DOI:** 10.3779/j.issn.1009-3419.2021.101.46

**Published:** 2022-02-20

**Authors:** 欣龙 刘, 滕 孙, 涛 洪, 延亮 袁, 昊 张

**Affiliations:** 1 221006 徐州，徐州医科大学附属医院胸外科 Department of Thoracic Surgery, Affiliated Hospital of Xuzhou Medical University, Xuzhou 221006, China; 2 221006 徐州，徐州医科大学第一临床医学院胸外科实验室 Thoracic Surgery Laboratory, The First College of Clinical Medicine, Xuzhou Medical University, Xuzhou 221006, China

**Keywords:** 肺肿瘤, 中央型肺癌, 新辅助免疫治疗, 达芬奇机器人, 支气管袖状切除, Lung neoplasms, Central lung cancer, Neoadjuvant immunotherapy, Da Vinci Xi robot system, Bronchial sleeve resection

## Abstract

**背景与目的:**

免疫新辅助为局部晚期肺癌治疗打开新局面。本研究探讨局部晚期中央型非小细胞肺癌（non-small cell lung cancer, NSCLC）患者在化疗联合免疫新辅助治疗后机器人辅助支气管袖状切除的安全性与可行性。

**方法:**

回顾性分析2020年8月-2021年2月接受化疗联合免疫新辅助治疗后支气管袖状切除的13例局部晚期中央型NSCLC患者的临床资料，患者依据手术方式不同分为开胸手术（thoracotomy bronchial sleeve resection, TBSR）组和机器人辅助手术（robot-assisted bronchial sleeve resection, RABSR）组，比较两组患者肿瘤学特征、术中和术后的资料。

**结果:**

两组患者手术顺利，术后病理证实均实现肿瘤R0切除，RABSR组患者无中转开胸。TBSR组中患者影像学部分缓解（partial remission, PR）率为71.43%，主要病理缓解（major pathological remissions, MPR）率为42.86%，完全病理缓解（complete pathological response, pCR）率为28.57%，RABSR组分别为66.67%、50.00%和33.33%。两组患者平均手术时长、淋巴结清扫数目、术中平均出血量、术后平均引流时间、术后住院时间无明显差异，但是RABSR组支气管吻合时间相对较短。两组患者均预后良好，顺利出院，术后90 d死亡率为0。

**结论:**

局部晚期中央型NSCLC患者经过化疗联合免疫新辅助治疗后可实现缩瘤降期、增加R0切除率，支气管袖状切除安全可行。在遵循手术安全性和实现肿瘤R0切除两个原则的前提下可优先选择机器人行袖状切除。

## Abstract (en)

**Background and objective:**

Immunoneoadjuvant therapy opens a new prospect for local advanced lung cancer. The aim of our study was to explore the safety and feasibility of robotic-assisted bronchial sleeve resection in patients with locally advanced non-small cell lung cancer (NSCLC) after neoadjuvant chemoimmunotherapy.

**Methods:**

Data of 13 patients with locally advanced NSCLC that underwent bronchial sleeve resection after neoadjuvant chemoimmunotherapy during August 2020 and February 2021 were retrospectively included. According to the surgical methods, patients were divided into thoracotomy bronchial sleeve resection (TBSR) group and robot-assisted bronchial sleeve resection (RABSR) group. Oncology, intraoperative, and postoperative data in the two groups were compared.

**Results:**

The two groups of patients operated smoothly, the postoperative pathology confirmed that all the tumor lesions achieved R0 resection, and RABSR group no patient was transferred to thoracotomy during surgery. Partial remission (PR) rate and major pathological remissions (MPR) rate of patients in the TBSR group were 71.43% and 42.86%, respectively. Complete pathological response (pCR) was 28.57%. They were 66.67%, 50.00% and 33.33% in RABSR group, respectively. There were no significant differences in operative duration, number of lymph nodes dissected, intraoperative blood loss, postoperative drainage time and postoperative hospital stay between the two groups, but the bronchial anastomosis time of RABSR group was relatively short. Both groups of patients had a good prognosis. Successfully discharged from the hospital and post-operative 90-d mortality rate was 0.

**Conclusion:**

In patients with locally advanced central NSCLC after neoadjuvant chemoimmunotherapy can achieve the tumor reduction, tumor stage decline and increase the R0 resection rate, bronchial sleeve resection is safe and feasible. Under the premise of following the two principles of surgical safety and realizing the tumor R0 resection, robot-assisted bronchial sleeve resection can be preferred.

2020年世界卫生组织国际癌症研究机构（International Agency for Research on Cancer, IARC）发布数据全球癌症死亡病例996万例，肺癌死亡180万例，远超其他癌症类型，居癌症死亡人数第一位。在中国，肺癌仍然是癌症新发病例的首位，非小细胞肺癌（non-small cell lung cancer, NSCLC）是肺癌最常见的类型^[1-3]^。近年由于磨玻璃结节（ground glass oracity, GGO）发病率高、手术切除率高以及预后更好^[4]^，外科医生对GGO更为关注，但对于局部晚期中央型NSCLC的治疗同样不能忽视，此类患者肿瘤位于肺门，通常直接手术需要进行双肺或全肺切除甚至难以切除，患者术后生活质量差、生存率低^[5]^。局部晚期中央型NSCLC患者行新辅助治疗后可缩小切除范围，提高肿瘤切除率，提升患者生活质量^[6]^。

近年免疫治疗兴起并在肺癌领域成为研究热点，为局部晚期肺癌的新辅助治疗打开新局面。CheckMate-159研究^[7]^指出，患者在新辅助免疫治疗过程中没有发生免疫治疗相关的手术延迟，23%的患者出现了免疫治疗相关不良反应，5%的患者为3级-4级不良事件。单臂Ⅰ期临床研究MK3475-223中^[8]^，患者接受2个周期帕博利珠单抗治疗，治疗期间未发生与新辅助治疗相关的手术延迟，也未发生手术相关并发症。NADIM研究^[9]^中46例可手术切除的IIIa期NSCLC肺癌患者，接受纳武利尤单抗、紫杉醇和卡铂的新辅助治疗后，最终41例患者完成手术，均实现肿瘤R0切除，主要病理缓解（major pathological remissions, MPR）率达83%，完全病理缓解（complete pathological response, pCR）率为71%，显示出化疗联合免疫新辅助治疗的强大优势。2020年中国《非小细胞肺癌新辅助免疫治疗的专家共识》指出^[10]^，对于局部晚期NSCLC患者可行化疗联合免疫新辅助治疗。

既往对于中央型NSCLC患者常需进行全肺切除手术，术后患者肺功能损失严重，尤其是对于老年人或者肺功能储备差的患者，全肺切除后生活质量显著下降^[11-13]^。袖状肺叶切除术现在被广泛认为是一种安全可靠的手术方式，可以完全切除侵入中心结构的肿瘤，相比全肺切除术可获得更好的短期恢复结果和长期生存结果^[14]^。胸腔镜手术已在胸外科常规应用，具有创伤小、恢复快的优势，但是胸腔镜操作灵活性受限，尤其是在袖状肺叶切除术中进行支气管吻合时操作难度较大，往往需要经验丰富的医生才能完成^[15]^。达芬奇机器人操作系统的出现极大地改变了这一现状，得益于三维成像技术、机械手臂的自由度和灵活性，术者更容易在狭小空间进行精细的吻合操作，保证吻合精准并且可以缩短手术时间^[16, 17]^。2019年青岛大学附属医院矫文捷教授团队^[18]^发表了迄今为止最大的单中心回顾性研究，证明在把握好手术适应证前提下，机器人辅助袖状肺叶切除术治疗中央型肺癌安全可行。

部分局部晚期中央型肺癌患者因支气管、血管条件欠佳或纵隔淋巴结肿大不具备直接袖状切除条件，化疗联合免疫新辅助治疗可使患者肿瘤缩小、病理分期下降，为此类患者手术创造可能。查阅数据库发现，化疗联合免疫新辅助治疗后支气管袖状切除术相关报道少见，尚无机器人手术应用于此类患者的报道。本研究总结本中心进行的13例NSCLC患者化疗联合免疫新辅助治疗后支气管袖状切除患者的病例资料，评估化疗联合免疫新辅助治疗后行开胸与机器人辅助支气管袖状切除术的患者在手术安全性、肿瘤病灶切除、支气管吻合操作难易及吻合精确性方面的差异，探讨局部晚期中央型NSCLC患者在化疗联合免疫新辅助治疗后机器人辅助支气管袖状切除术的安全性及可行性。

## 1 资料与方法

### 1.1 筛选目标人群

该研究根据《赫尔辛基宣言》（2013年修订）进行。研究方案和方法由徐州医科大学附属医院伦理委员会评审。收集2020年8月-2021年2月连续入住徐州医科大学附属医院胸外科的支气管袖状切除术后所有经病理证实的NSCLC患者的数据资料，筛选经化疗联合免疫新辅助治疗后支气管袖状切除的局部晚期中央型NSCLC患者。

### 1.2 一般资料

本研究中的患者术前均通过支气管镜活检明确病理类型为NSCLC，肿瘤位于肺门支气管、可伴随纵隔或支气管周围淋巴结转移，但无远处器官转移。对所有患者进行胸部增强计算机断层扫描（computer tomography, CT）、颅脑核磁共振（magnetic resonance imaging, MRI）、肺功能、心脏彩超、腹部彩超、全身骨骼显像（emission computed tomography, ECT）和血液学等方面的评估，部分患者检查正电子发射计算机断层显像（positron emission tomography- CT, PET-CT）。本研究已获徐州医科大学附属医院伦理委员会批准并获得所有患者的书面知情同意书。

### 1.3 结局指标评价

共收集13例患者资料，患者依据手术方式分为两组：开胸手术（thoracotomy bronchial sleeve resection, TBSR）组（*n*=7，男5例、女2例）和机器人辅助手术（robot-assisted bronchial sleeve resection, RABSR）组（*n*=6，男5例、女1例）。收集两组患者资料：①一般资料：年龄、性别、肿瘤大小、肿瘤部位、吸烟史等；②术中资料：手术时间、淋巴结清扫数量、支气管吻合时间、术中出血量等；③术后资料：术后胸腔引流量、引流管留置时间、手术并发症、术后住院时间等；④病理学评估：病理检查结果。手术时间为患者手术切皮开始至皮肤缝合结束。所有接受肺叶切除术的患者均常规行系统淋巴结清扫，右侧清扫2、3、4、7、8、9、10、11组淋巴结；左侧清扫4、5、6、7、8、9、10、11组淋巴结。根据美国癌症联合委员会和国际肺癌研究协会的指南定义淋巴结站。术后并发症由临床观察、常规检查及影像复查确诊。术后第2天行床旁X线检查，无术后肺漏气、炎症、胸腔积液等并发症，且24 h引流量≤150 mL的患者拔出胸腔引流管^[19]^。病情稳定的患者于拔管后第2天出院。

### 1.4 治疗方案

13例患者均进行化疗联合免疫新辅助治疗，治疗方案为紫杉醇+顺铂（卡铂）+帕博丽珠单抗（或信迪利单抗或卡瑞丽珠单抗）见表 1，新辅助治疗周期为2个-4个。新辅助治疗方案由肿瘤科、呼吸科、胸外科多学科诊疗团队（multiple disciplinary team, MDT）讨论并和患者共同商定，新辅助治疗在呼吸科或肿瘤内科完成。新辅助治疗期间无患者出现3级-4级严重不良反应，未发生与新辅助治疗相关的手术延迟。每2个周期进行一次胸部CT检查评估新辅助治疗效果，手术时机结合治疗前后影像学资料、支气管镜检查结果综合考虑。手术方式选择上优先考虑应用机器人手术，在全身麻醉后经观察孔（腋中线第7肋间）进行胸腔镜探查，若发现肺门结构固定、门钉淋巴结等难以机器人操作的病例，选择开胸手术。最终6例患者选择机器人辅助支气管袖状切除术，7例患者选择开胸支气管袖状切除术。袖状肺叶切除术包括肺叶切除术伴完全环形支气管袖状切除术和系统性肺门及纵隔淋巴结切除术。所有机器人手术均使用达芬奇Xi手术系统进行。由同一手术小组对每位患者进行手术。

**表 1:** TBSR组和RABSR组治疗策略 Treatment strategies of TBSR group and RABSR group

Treatment strategies	TBSR group (*n*=7)	RABSR group (*n*=6)
Chemoimmunotherapy		
Paclitaxel+cisplatin (carboplatin)	7	6
Anti-PD-1		
Pembrolizumab	2	1
Sintilimab	1	2
Camrelizumab	4	3
Middle neoadjuvant therapy cycles	3 (2-4)	3 (2-4)
Interval between neoadjuvant therapy and surgery (d)	36 (30-54)	34 (28-48)
TBSR: thoracotomy bronchial sleeve resection; RABSR: robot-assisted bronchial sleeve resection; PD-1: programmed death protein-1		

### 1.5 技术

#### 1.5.1 机器人手术

患者全身麻醉双腔气管插管后取健侧卧位，双上肢均稍屈向头侧伸展，充分暴露手术视野，并保证机械臂的最大活动度，患者固定后，将手术床胸段以下向床体折叠15°（呈折刀位），用于拉宽肋间隙利于手术操作。手术区域常规消毒铺巾。达芬奇机器人手术系统，光源孔通常选在腋中线第7肋间，镜头30°向下，第1、第2臂操作孔根据左右肺不同可选腋前线稍偏前与肩胛下角线稍偏后的第6肋间和第7肋间，两臂间距8 cm左右，辅助操作孔首选第4肋间隙腋前线处，以对向肺门为原则，根据患者体型和肿瘤位置调整切口位置。探查胸腔内的病变情况，分离下肺韧带，清扫隆突下淋巴结。分离叶间裂，游离分离肺动脉及静脉各分支，通过辅助操作孔自动切割闭合器离断。分离支气管，游离支气管旁结缔组织，离断病变气管，并通过辅助孔协助控制台的外科医生进行支气管吻合。支气管吻合方式5例患者选择连续缝合，仅1例患者因右主支气管埋于奇静脉深处连续缝合困难，使用半连续缝合^[16, 20]^。术中严密止血，生理盐水冲洗术野，并吸痰、鼓肺，确认吻合口无漏气。放置胸腔引流管一根，缝合切口。

#### 1.5.2 开胸手术

患者全身麻醉双腔气管插管后取健侧卧位，双上肢均稍屈向头侧伸展，充分暴露手术视野，患者固定后，手术区域常规消毒铺巾。切口沿第4或第5肋间由胸骨侧缘向后上达腋中线（女性沿乳房下缘弧形切口），切断部分胸大肌和前锯肌，暴露肋骨和肋间隙，切开肋间肌，剪开胸膜，显露胸腔。分离下肺韧带，按常规处理方法肺动脉和静脉，完全分开斜裂和或水平裂，解剖出左右主支气管，橡皮条围绕并牵引。两环状软骨之间分别切断主支气管，支气管切缘的近端（主支气管）和远端（中间支气管）均送病理科行冰冻切片检查，病理阴性时，行支气管吻合，支气管吻合方式为连续缝。术中严密止血，生理盐水冲洗术野，并吸痰、鼓肺，确认吻合口无漏气。放置胸腔引流管一根，缝合切口。

### 1.6 统计学方法

所有数据采用SPSS 25.0软件处理。连续数据作为均数±标准差（Mean±SD）表示，并用*t*检验对数据进行分析。分类变量以患者的计数和百分比表示，并采用λ^2^或*Fisher*的精确测试进行比较。以*P* < 0.05为差异有统计学意义。

## 2 结果

### 2.1 患者资料

归纳总结两组患者年龄、肿瘤大小、吸烟史、肿瘤部位、肿瘤分期等一般资料，见表 2。TBSR组患者肿瘤位置：左肺上叶1例、左肺下叶3例、右肺上叶2例、右肺中叶1例，RABSR组患者肿瘤位置：左肺下叶1例、右肺上叶1例、右肺中叶1例、右肺下叶3例。13例患者中，IIa期患者2例（15.38%），IIb期患者1例（7.69%），IIIa期患者6例（46.16%），IIIb期患者3例（23.08%），IIIc期患者1例（7.69%）。支气管吻合方式：TBSR组均采取连续缝合，RABSR组5例采取连续缝合，1例采取半连续缝合。

**表 2:** 患者基线资料 Patient baseline characters

Characters	TBSR group (*n*=7)	RABSR group (*n*=7)
Age (yr)	62.50 (57-83)	61.33 (51-67)
Gender		
Male	5 (38.46%)	6 (46.14%)
Female	2 (15.38%)	0
Tumor position		
Upper left lung	1 (7.69%)	0
Lower left lung	3 (23.07%)	1 (7.69%)
Upper right lung	2 (15.38%)	1 (7.69%)
Middle right lung	1 (7.69%)	1 (7.69%)
Lower right lung	0	3 (23.07%)
Tumor diameter (cm, range)	6.71 (3-15)	6.78 (3-13)
Smoking history		
Former/current	4 (30.77%)	4 (30.77%)
Never	3 (23.07%)	2 (15.38%)
Diabetes	0	0
Clinical stages		
IIa			
T2bN0	1 (7.69%)	1 (7.69%)
IIb			
T2bN1	0	1 (7.69%)
IIIa			
T1cN2 T2aN2 T2bN2 T4N0	0 1 (7.69%) 1 (7.69%) 1 (7.69%)	1 (7.69%) 1 (7.69%) 1 (7.69%) 0
IIIb			
T3N2 T4N2	1 (7.69%) 1 (7.69%)	1 (7.69%) 0
IIIc			
T4N3	1 (7.69%)	0

### 2.2 围手术资料

TBSR组与RABSR组患者围手术期资料见表 3。两组患者术中与术后结果对比，平均手术时长：TBSR组为149.71（110-175）min，RABSR组为131.67（120-150）min；淋巴结清扫数目：TBSR组为（17.29±1.89）枚，RABSR组为（18.17±1.17）枚；支气管吻合时间：TBSR组为23.86（21-28）min，RABSR组为18.50（16-22）min；术中平均出血量：TBSR组为94.28（50-200）mL，RABSR组为79.16（75-110）mL；术后平均引流时间：TBSR组为（5.28±1.38）d，RABSR组为（3.83±2.07）d；术后住院时间：TBSR组为（6.29±2.75）d，RABSR组为（5.62±1.47）d。支气管吻合时间：TBSR组为23.86（21-28）min，RABSR组为18.50（16-22）min，差异有统计学意义（*P* < 0.05），表明机器人在进行支气管吻合时较开胸手术所需时间更短，吻合速度更快，机器人操作系统在进行支气管吻合时更具优势。

**表 3:** TBSR组和RABSR组术中与术后资料 Intraoperative and postoperative data of TBSR group and RABSR group

	TBSR group	RABSR group
Operation time (min)	149.71 (110-175)	131.67 (120-150)
Number of lymph node	17.29±1.89	18.17±1.17
Bleeding (mL)	94.28 (50-200)	79.16 (75-110)
Bronchial anastomosis (min)	23.86 (21-28)	18.50 (16-22)
Drainage time (d)	5.28±1.38	3.83±1.32
Postoperative hospitalization time (d)	6.29±2.75	5.62±1.47

### 2.3 手术结局

在TBSR组中，5/7（71.43%）患者获得影像学PR，3/7（42.86%）患者获得MPR，2/7（28.57%）患者获得pCR；在RABSR组中，4/6（66.67%）患者获得影像学PR，3/6（50.00%）患者获得MPR，2/6（33.33%）患者获得pCR。所有患者术后病理学检查均显示支气管切缘未见肿瘤细胞残留，肿瘤病灶均实现R0切除。

### 2.4 安全性

13例患者中有3例患者出现术后并发症，TBSR组1例患者发生肺部感染，RABSR组2例患者分别为肺部感染和吻合口梗阻（梗阻内容物为痰液）。13例患者吻合口方式选择半连续或者连续缝合，仅机器人手术组1例患者进行半连续缝合，其余患者支气管吻合方式均为连续缝合。两组患者均顺利完成手术，无患者发生手术延迟，RABSR组无患者中转开胸，所有患者术后恢复良好，最终康复出院。术后90 d死亡率为0。

## 3 讨论

研究^[21, 22]^显示免疫新辅助治疗为一部分无法直接手术的局部晚期NSCLC患者创造了手术机会。上海胸科医院罗清泉教授团队的两项研究表明，可切除的NSCLC患者在接受化疗联合免疫新辅助治疗后肺部手术安全可行。广州医科大学附属医院李树本教授团队及天津医科大学肿瘤医院尤健教授团队^[23, 24]^证实中央型肺癌患者接受化疗联合免疫新辅助治疗后行支气管袖状切除安全有效。但尚无局部晚期中央型NSCLC患者接受化疗联合免疫治疗新辅助后进行机器人支气管袖状切除的病例报道。本研究总结了13例局部晚期中央型NSCLC患者接受化疗联合免疫新辅助治疗后进行开胸和机器人支气管袖状切除术的结果。13例患者在新辅助治疗前评估直接手术切除困难、难以实现R0切除或需要进行全肺切除。所有患者术前均接受化疗联合免疫新辅助治疗，新辅助治疗期间无患者因发生相关不良发应提前终止治疗。在完成新辅助治疗后无患者发生手术延迟，手术均完成根治性切除，术后3例患者出现并发症，预后良好，顺利出院，术后90 d内死亡率为0。13例患者中6例实现MPR（MPR率为46.2%），4例实现pCR（pCR率为30.7%），与梁恒瑞^[23]^、沈迪建^[6]^，康进^[25]^等的研究结果一致。本研究资料分析表明局部晚期中央型NSCLC患者接受化疗联合免疫新辅助治疗后行支气管袖状切除术是一种安全可行的治疗方案。

2020年中国《非小细胞肺癌新辅助免疫治疗的专家共识》指出暂无证据表明化疗联合免疫新辅助治疗会影响手术操作或安全性。根据本研究实践总结，我们尝试从两个维度对手术难度进行分析。一方面，部分患者肿瘤病灶过大，几乎没有手术空间，直接进行手术切除难度巨大，但新辅助治疗后肿瘤缩小，创造了手术空间，降低了手术难度；另一方面，经过化疗联合免疫新辅助治疗后，大部分患者血管鞘膜增厚，肿瘤周围组织水肿，毛细血管脆性增加，淋巴结皱缩导致组织间隙变窄，导致手术操作难度增加，术中出血风险增加。这些改变与既往报道^[6]^情况相似，曾行化疗联合免疫新辅助治疗后手术的外科医生应该都有此体会。

既往研究^[18]^表明对于可手术的中央型肺癌行支气管袖状切除的病例，相比开胸或胸腔镜手术，机器人手术术中出血更少、胸腔引流管拔除更早、支气管吻合和手术时间更短，以及相似的肿瘤学效果。但根据本研究实践总结，基于化疗联合免疫新辅助治疗可能造成的潜在组织学改变，对于新辅助后才具备手术机会的患者，若行支气管袖状切除术，应结合患者新辅助治疗前后的影像学资料、支气管镜检查结果，对比新辅助治疗前后肿瘤大小变化、肿瘤退缩程度，缩小肿瘤或淋巴结与支气管或肺动脉之间的关系，在术前仔细评估手术风险及难度选择性的采用开胸或机器人手术；术前亦可通过腔镜打孔探查肺门结构是否纤维化严重导致固定、是否有门钉淋巴结等进行综合评估决定。我们考虑对于化疗联合免疫新辅助治疗后的患者，应在充分考虑手术安全性和实现肿瘤R0切除这两个原则的前提下选择机器人或开胸手术进行支气管袖状切除。

基于以上原则考量，本研究最终有7例患者选择开胸手术，6例行机器人手术。回顾性分析患者手术资料显示，平均手术时长：TBSR组为149.71（110-175）min，RABSR组为131.67（120-150）min；引流管留置时间：TBSR组为（5.28±1.38）d，RABSR组为（3.83±2.07）d；术后住院时间：TBSR组为（6.29±2.75）d，RABSR组为（5.62±1.47）d，两组患者存在差异，但是由于患者数量少，手术方式选择存在偏倚，因此两组患者手术资料我们仅进行了分析、归纳、总结，未进行统计学分析。虽然本研究对于手术条件不好的患者选择了开胸手术，但在肿瘤切除后，开胸手术与机器人手术面临的支气管吻合条件相似，因此我们考虑这种情况下支气管吻合时间具备可比性，因此我们比较了两组在支气管吻合时间的区别：TBSR组为23.86（21-28）min，RABSR组为18.50（16-22）min，两组患者支气管吻合时间存在差异（*P* < 0.05），统计学分析结果表明机器人手术方式下实现了更快更精准的支气管吻合。因为机器人系统的三维立体视野以及具有7个自由度的活动关节的专用器械，因此机器人下行支气管吻合更加确切，同时机械臂拥有更好的稳定性，有效提升了术者操作速度和精确性。此外，有研究^[26]^指出，机器人手术操作系统在淋巴结清扫数目与淋巴结清扫站数方面更具有优势，得益于机械臂的灵活性以及术者对手术更高的掌控性，使术者更加愿意尝试切除复杂位置的淋巴结，并且术者通过3D立体视野可以更加细致地观察胸腔内结构进而更加精细地处理淋巴结及各处组织。在我们的研究中，TBSR组的淋巴结清扫数目平均17.29枚；RABSR组为18.17枚，机器人手术在淋巴结清扫数目及站数上与以往报道^[26]^一致。因此，我们的体会是对于化疗联合免疫新辅助治疗后的患者，我们在手术方式选择上应优先选择机器人手术等微创手段，但也应结合实际情况不强求和拘泥于微创。

我们的研究尚有不足之处，首先这是一篇回顾性研究，纳入病例数相对较少，未来需要更多的病例进行补充；其次，我们仅总结了新辅助化疗免疫治疗后开胸与机器人支气管袖状切除，未能把胸腔镜下支气管袖状切除术纳入此次研究中进行归纳、分析，我们将在下一步工作中纳入更多的病例进一步研究。此外，本研究随访时间较短，新辅助化疗联合免疫治疗后机器人支气管袖状切除的长期益处尚未清楚，我们将进行更长期的随访观察进一步明确。

总之，对于局部晚期中央型NSCLC患者，本研究显示化疗联合免疫新辅助治疗可使肿瘤体积缩小，降低肿瘤分期，增加R0切除率，为一部分支气管或血管条件不好，难以直接手术的患者争取了手术机会，对于此类患者行支气管袖状切除是安全可行的。化疗联合免疫新辅助治疗后，我们应遵循手术安全性和实现肿瘤R0切除这两个原则，并结合患者新辅助治疗前后的各种资料进行具体评估选择开胸或机器人手术进行支气管袖状切除。
